# Effect of 6-Shogaol on the Glucose Uptake and Survival of HT1080 Fibrosarcoma Cells

**DOI:** 10.3390/ph12030131

**Published:** 2019-09-09

**Authors:** Angie C. Romero-Arias, Luis G. Sequeda-Castañeda, Andres F. Aristizábal-Pachón, Ludis Morales

**Affiliations:** 1 Department of Nutrition and Biochemistry, School of Sciences, Pontificia Universidad Javeriana, Bogotá 110231, Colombia; 2 Department of Chemistry, School of Sciences, Pontificia Universidad Javeriana, Bogotá 110231, Colombia

**Keywords:** 6-shogaol, HT1080, ROS, survival, glucose

## Abstract

Ginger is a plant that is native to southern China. In the last decade and research on the components of ginger has significantly increased; of these components, 6-shogaol exhibits the greatest potential antitumor capacity. However, the molecular mechanism through which 6-shogaol exerts its effects has not yet been elucidated. In this study, the effect of 6-shogaol on tumor cells that were derived from human fibrosarcoma (HT1080) was evaluated. Cell viability was determined by a (3-(4,5-dimethylthiazol-2-yl)-2,5-diphenyltetrazolium bromide) MTT assay testing different concentrations of 6-shogaol (2.5–150 μM). Subsequently, the effect of 6-shogaol on reactive oxygen species (ROS) production, glucose uptake, and protein expression of the signaling pathway phosphatase and tensin homolog/ protein kinase B /mammalian target of rapamycin (PTEN/Akt/mTOR) was measured. 6-Shogaol reduced the viability of the tumor cells and caused an increase in ROS production, which was attenuated with the addition of *N*-acetylcysteine, and the recovery of cell viability was observed. The increase in ROS production in response to 6-shogaol was associated with cell death. Similarly, glucose uptake decreased with incremental concentrations of 6-shogaol, and an increase in the expression of mTOR-p and Akt-p proteins was observed; PTEN was active in all the treatments with 6-shogaol. Thus, the results suggest that cells activate uncontrolled signaling pathways, such as phosphoinositide 3-kinase (PI3K)/Akt/mTOR, among other alternative mechanisms of metabolic modulation and of survival in order to counteract the pro-oxidant effect of 6-shogaol and the decrease in glucose uptake. Interestingly, a differential response was observed when non-cancerous cells were treated with 6-shogaol.

## 1. Introduction

Ginger (*Zingiber officinale* Roscoe, Zingiberaceae) has been used for thousands of years as a spice, and it has been considered to be an important ingredient in traditional Chinese medicine for the treatment of certain diseases, such as diabetes, cardiovascular diseases, rheumatism, and cancer [1,2,3,4,5,6,7,8]. In the last decade, there has also been progress in the study of other biological properties of ginger, such as its antifungal and antimicrobial capacity [9]. Interestingly, many studies have focused on the antioxidant, anti-inflammatory, and antitumor capacity, however, the molecular mechanisms through which their action is exerted are not yet known.

The nutritional value of ginger can be attributed to a variety of bioactive compounds, including zingiberene, gingerols, and shogaols [1,10]. Gingerols and shogaols are groups of volatile phenolic compounds, and they are mainly responsible for the pungency of the rhizome [11]. It has been reported that 6-shogaol has better biological activities than 6-gingerol, especially in relation to antitumor activity [8,12].

Cancer is a pathology of high global prevalence and rapid growth. It has been reported that, in various cancer models, ginger extract has the ability to decrease cell survival, increase ROS production, stimulate hyperpolarization of the mitochondrial membrane, and mediate the inactivation of the Akt protein [13,14]. However, given the amount of bioactive compounds in the extract, it is relevant to inquire about the effect of its components and how these can promote intracellular processes that lead to cell death, or better yet, regarding the synergistic effect between the different components that can be exerted on the cells. 

In this sense, the objective of this study was to evaluate the effect of 6-shogaol—as one of the components with greater activity—on glucose uptake and tumor cell survival by evaluating the production of ROS and some modulators of one of the canonical pathways of cell survival.

## 2. Results

### 2.1. 6-Shogaol Induces Cell Death in Fibrosarcoma Cells

**Figure 1:**
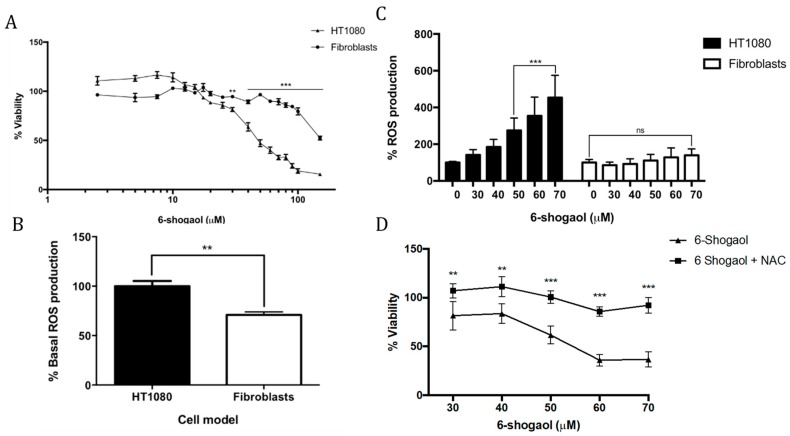
6-Shogaol effects on cancer and normal cells. (**A**) HT1080 and fibroblasts derived human periodontal ligament (HPdLF) cell viability; (**B**) Basal reactive oxygen species (ROS) production in HT1080 and HPdLF cells; (**C**) Effect of 6-shogaol exposure in reactive oxygen species (ROS) production in HT1080 and HPdLF cells; and, (**D**) HT1080 and HPdLF cell viability co-treated with N-acetyl cisteine (NAC) and 6-shogaol. ** *p* = 0.01; *** *p* = 0.001.

6-Shogaol (2.5–150 μM) decreases HT1080 cell viability in a dose-dependent manner. The half maximal inhibitory concentration (IC_50_) of 6-shogaol in HT1080 cells was 52.8 μM. Low concentrations of 6-shogaol exhibited effects on the cell viability of the HT1080 tumor model. From 30 μM, a clear decrease in cell viability was observed, even below 80%. No cells survived at 150 μM 6-shogaol. Fibroblasts that were derived from human periodontal ligament (HPdLF) exhibited greater resistance to treatment with 6-shogaol as compared to the HT1080 cells (*p* = 0.0058). HPdLF decreased its viability from 70 μM 6-shogaol (Figure 1A).

### 2.2. NAC Attenuates the Pro-Oxidant Effect of 6-Shogaol in Tumoral Model Cells (HT1080)

HT1080 cells showed a significantly higher basal ROS production (*p* = 0.0011) than fibroblasts that were derived from the periodontal ligament (Figure 1B). According to other research groups, tumoral cells show higher ROS production due to their genetic, metabolic, and tumor microenvironment alterations, which allows them to increase their growth, proliferation, and survival [15].

To evaluate cell dynamics in response to treatment with 6-shogaol, concentrations of 30–70 μM were used, which resulted in a significant decrease in cell viability when applied in the tumor model. An increase in dose-dependent ROS production was observed in HT1080 cells, which was significant at 50 μM 6-shogaol (*p* = 0.0001). The fibroblasts did not show alterations following treatment with 6-shogaol. Importantly, it can be concluded that, relative to ROS production in response to treatment with 6-shogaol, a different effect is observed between the tumor and non-tumor cells (Figure 1C). When treating both cell models with 5 mM NAC (N-Acetyl Cysteine) in the presence of 70 μM 6-shogaol, a statistically significant decrease in ROS production was observed (*p* = 0.001 HT1080) (*p* = 0.0029 HPdLF). This allowed us to conclude that the increase in ROS production was due to the effect of 6-shogaol.

Finally, the cell viability of the tumor model was evaluated under treatment with 6-shogaol (30–70 μM) and joint treatment with NAC (5 mM). Cells only treated with 6-shogaol showed decreased viability at all concentrations (Figure 1D), whereas cells that were treated together with NAC and 6-shogaol showed an attenuation of the cytotoxic effect of 6-shogaol (*p* = 0.0091). Therefore, we concluded that cell death generated by 6-shogaol is associated with an increase in ROS production.

### 2.3. 6-Shogaol Decreases Glucose Uptake in HT1080 Cells

The assay results showed that basal glucose uptake by the tumoral cell model was greater than for normal fibroblasts (*p* = 0.0114), which can be explained by the high metabolic activity and high energy demand of tumoral cells for the maintenance of their proliferation [16]. 

**Figure 2:**
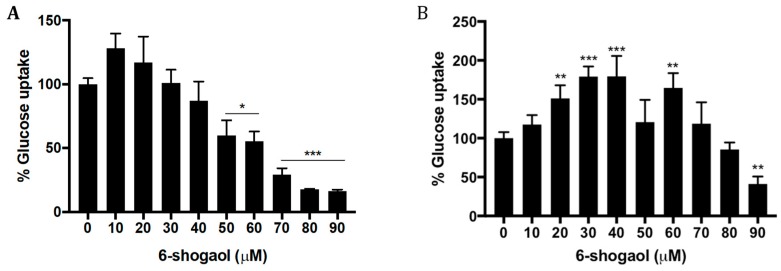
Effect of 6-shogaol exposure on glucose uptake. Glucose uptake in (**A**) HT1080 and (**B**) HPdLF cells. * *p* = 0.05; ** *p* = 0.01; *** *p* = 0.001.

In response to treatment with 10–90 μM 6-shogaol, it was observed that the uptake of glucose by the tumor cells significantly decreased from 50 μM (*p* = 0.0259, Figure 2A). By contrast, non-tumoral cells, in response to 6-shogaol, showed an increase in glucose uptake, which was significant from 20 to 40 μM (*p* = 0.0201), however, from 60 μM 6-shogaol, a progressive decrease in glucose uptake was observed with increasing 6-shogaol concentration (*p* = 0.0065, Figure 2B).

### 2.4. 6-Shogaol Produced a Survival Decrease through PI3K/AKt/mTOR Modulation on HT1080 Cells

**Figure 3:**
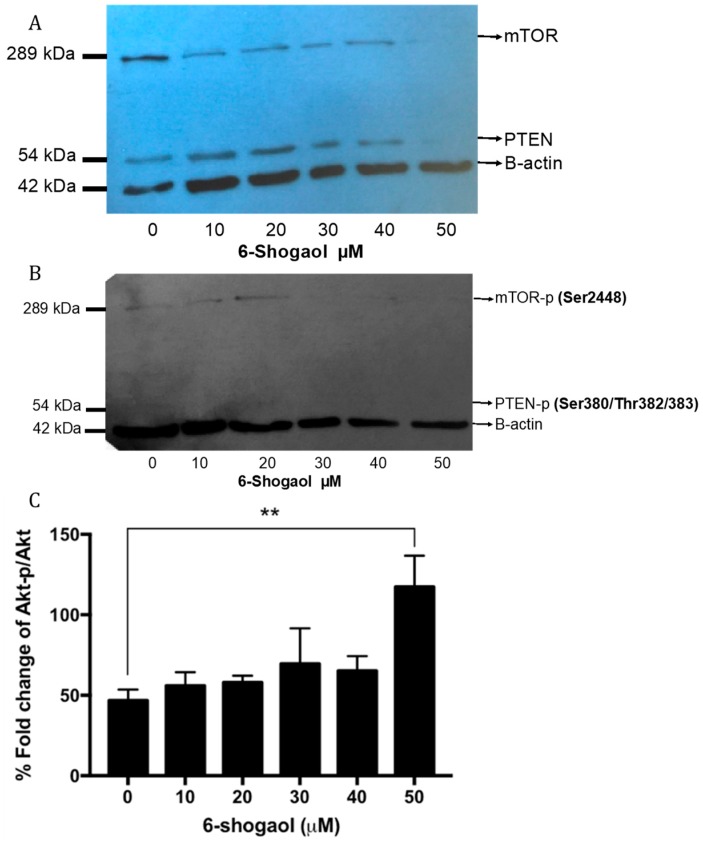
The effect of 6-shogaol on mammalian target of rapamycin (mTOR) and protein kinase B (AKT) expression. (**A**) mTOR and Phosphatase and tensin homolog (PTEN) proteins expression; (**B**) mTOR and PTEN protein activation; (**C**) Akt protein activation. ** *p* = 0.01.

The expression and activation of the Akt, PTEN, and mTOR proteins were evaluated by Western blot and flow cytometry in order to advance the mechanism of action exerted by 6-shogaol. It was observed that HT1080 cells under 6-shogaol exposure of up to 40 μM maintained their expression of PTEN and mTOR. However, at higher concentrations (> 40 μM), there was a decrease in the expression of these proteins (Figure 3A). 

It was observed that 6-shogaol (10 and 50 μM) induced phosphorylation of the mTOR protein, whereas PTEN was not observed to be phosphorylated at any of its three activation sites; this suggests that PTEN could be in its active (dephosphorylated) form (Figure 3B).

As with the mTOR and PTEN proteins, the expression of Akt and Akt-p in response to treatment with 6-shogaol did not show significant changes at low concentrations. However, phosphorylation of Akt significantly increased at 50 μM 6-shogaol (*p* = 0.0091) (Figure 3C).

## 3. Discussion

The differential effects of 6-shogaol on normal and tumor cell lines have already been described, however the cause of this compound having this differential effect is still unknown. It is thought that the effect is related to the increased production of ROS induced by 6-shogaol, which acts as a pro-oxidant; it is likely that this effect is more effectively neutralized by non-tumor cells through more efficient endogenous antioxidant mechanisms. In addition, it can be considered that tumor cells already have a high amount of intracellular ROS, which can make them more sensitive to this type of insult, which compromises their viability.

It should be considered that 6-shogaol is not the only volatile compound with biological activities against cancer that is present in ginger. It is also known that 6-gingerol has similar antitumoral properties, but to a lesser degree [11].

**Figure 4:**
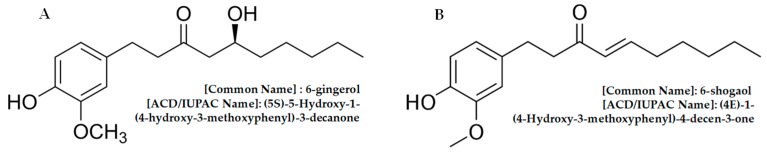
Chemical structures of (**A**) 6-gingerol and (**B**) 6-shogaol. Structures were made with free ChemSpider software [22].

The gingerols are present at higher concentrations in fresh root. The main isoform of gingerol has a double bond in carbon 6, and it is called 6-gingerol (Figure 4A). 6-Gingerol has several biological activities that have been described in the treatment of chronic diseases in humans and animal models [17]. Within these reported activities are anti-aging effects, which are related to the ability to inhibit vascular senescence that is regulated by the signaling pathway of mTOR [18], antitumor effects based on the ability to modulate some signaling pathways that are involved in apoptosis, regulation of the cell cycle, cytotoxic activity, and inhibition of angiogenesis [19].

It has been shown that 6-gingerol inhibits the progression of skin cancer induced by phorbol ester in addition to inhibiting metastasis in breast cancer (MDA-MB-231), induced apoptosis in prostate cancer (LNCaP), and it could inhibit tumor progression in melanomas and kidney cancer in murine models; there are few reports in relation to other types of cancer [20].

Shogaols (Figure 4B) are analogous compounds of gingerols; they are thermally labile and are formed by reversible reactions of dehydration of gingerol at high temperatures [10] or, in some cases, are the product of storage. 6-Shogaol has a higher biological activity when compared to 6-gingerol, and reports also mention a lower antioxidant activity when evaluated by the DPPH (2,2-diphenyl-1-picrylhydrazyl) and FRAP (ferric reducing ability of plasma) method [21]; therefore, in cell cultures, ROS production is increased to a greater extent in tumor models when treating with 6-shogaol compared to 6-gingerol [14].

Similarly, it has been reported that 6-shogaol has greater anti-inflammatory capacity than 6-gingerol, which inhibits the production of prostaglandin E2 (PGE2) as well as the expression of proinflammatory cytokines, such as interleukin 1β, tumor necrosis factor (TNF-α), p38, and nuclear factor kappa β (NF-kB). In addition, it inhibits the release of nitric oxide and the expression of the enzyme nitric oxide synthase in mouse microglia cells (BV-2) treated with lipopolysaccharide (LPS), primary cultures of glia, and in vivo models of neuroinflammation [23]. There are still no reports regarding studies with fibrosarcoma.

The cytotoxic effects of 6-shogaol have been described in several tumor lines and using different concentrations, such as in the case of HT-29 cells of colorectal cancer, whose viability was reduced by 50% when treated with 60 μM and they were completely inviable upon treatment with 80 μM [24]. Other tumor lines have shown greater sensitivity to 6-shogaol; among them are lung carcinoma (A549 cells) and breast cancer (MDA-MB-231 cells), which presented an IC_50_ of 29.6 and 22.1 μM, respectively, when treated with 6-shogaol for 48 h [25]. The difference in the IC_50_ between the tumor lines might be due to the histological differences of each of the cell lines, which affects their differential sensitivity to the compound. 

In the present work, the significant differences in viability that were observed between non-tumor cells (HPdLF) and the HT1080 line in response to 6-shogaol treatment (*p* = 0.0001) coincide with reports that were described in human gingival fibroblasts (HGF-1), where a 10% decrease in cell viability was observed when treated with upwards of 80 μM 6-shogaol [26].

The differential biological activities of 6-gingerol and 6-shogaol in the different cellular models are related to their structure. 6-Gingerol differs from 6-shogaol in its side chain, since it contains a β-hydroxy ketone, whereas 6-shogaol is a dehydration product of 6-gingerol, which in turn possesses a α,β-unsaturated ketone. The metabolism of 6-shogaol both outside and inside the cell continues to be studied. It is known that under conditions of heat or acidity outside the cell, the β-hydroxyl ketone of 6-gingerol is converted to an α,β-unsaturated ketone, which gives rise to the 6-shogaol molecule. Thus, the conjugation of the α-β-unsaturated ketone skeleton of 6-shogaol explains its better efficacy when compared to 6-gingerol in terms of antitumor capacity [27].

From advanced studies in mice and tumor cells, it is currently known that 6-shogaol is intracellularly metabolized into 13 metabolites (M1–M13). However, the mechanism of 6-shogaol uptaken by cells is unknown [28].

Studies of the SAR (structure–activity relationship) have shown that the free hydroxyl group of 6-gingerol contributes to its antitumor activity against breast cancer, and that the length of the aliphatic side chain increases this activity, since this chain loses activity when its length is reduced, as shown in the tumor models [29]. However, 6-shogaol has better biological activity against tumor cells and in murine models, which can be attributed to its main conjugated metabolite, cysteinyl-6-shogaol, which is a product of its pharmacokinetics and that enhances the ability of 6-shogaol to halt cell cycle progression, induce apoptosis, and inhibit cell growth through the modulation of cellular signaling of NF-kB and STAT3 [20].

In tumor cells, the presence of complex alterations, such as disruption of some metabolic pathways and the permanent activation of signaling pathways involved in survival, as well as the increase in nutrient consumption, can lead not only to a higher production of ROS, but also to a decrease in the ability to neutralize them effectively; therefore, the tumor cell exhibits a higher production of ROS. To counteract the effect of increased ROS on viability, recent studies have indicated that tumor cells develop alternative mechanisms of activation of antioxidant pathways, which are not sufficient when cells undergo additional pro-oxidant stimuli (6-shogaol). It is well known that increased ROS production in tumor cells induces the uncontrolled activation of the PI3K signaling pathway (PI3K/Akt/mTOR), in comparison with the response of normal cells. Consequently, the RAS pathway is activated, which leads to metabolic modulation and survival. Additionally, the resulting hyperactivation of HIF-α is also mediated by factors, such as VEGF, modular angiogenesis, and increased proliferation and cell migration, among others [30].

Similar results to those that were observed in the present work have also been described in other tumor models, such as mouse kidney epithelial cells (TMCK-1) and mesangial cells (MC) [31]. These also coincide with reports that were made in 2016 on colorectal cancer cells, where 5 μM 6-shogaol resulted in a doubling of ROS production as compared to the control, and at 20 μM, ROS production was up to four times higher than the control. This report also evaluated the activation of antioxidant enzymes, which was decreased with increasing 6-shogaol concentration [26].

Similarly, Akimoto et al. 2015 demonstrated that the pro-oxidant capacity of 6-shogaol and its effect on the reduction of cell viability can be maintained with the treatment of 10 mM NAC in a cellular model of PANC-1 (pancreatic tumor cells) [14]. It has been described that the pro-oxidant capacity of 6-shogaol in tumor cells has been associated with an α,β-unsaturated carbonyl group that participates in Fenton reactions and promotes the production of hydroxyl radicals that have a high affinity for DNA and some proteins, allowing for 6-shogaol to decrease the viability of this type of cell [26]. 

The effect of 6-shogaol on the increase of glucose uptake in non-tumor cell models was described for the first time in adipocytes (3T3-L1) and myotubules (C2C12), demonstrating that this compound generated a greater uptake in comparison with 6-gingerol and zingerone [32]. This effect was attributed to the increase in phosphorylation of AMPK, although it is likely that there are additional mechanisms that have not yet been studied. To our knowledge, there are no reports regarding the effect of 6-shogaol on glucose uptake in tumoral models; however, it is known that 6-gingerol, which is another of the pungent compounds of ginger, has the ability to increase glucose uptake in smooth muscle cells and diabetic obese mouse models through the increase in translocation of GLUT-4 in the surface of the cell membrane (Son, Miura, & Yagasaki, 2015) and in skeletal muscle cells (C2C12), which increases the phosphorylation of Akt [33].

The basal glucose uptake that was determined in both cell models showed that the tumor model captures more glucose than the non-tumor model (*p* = 0.0114); this can be explained by its high metabolic activity and its high energy demands for the maintenance of its proliferation rate (10). Additionally, the effect is characteristic of the so-called Warburg effect, which, with its modifications, has been described in recent literature reports as aerobic lactate genesis, characterized by a metabolic reprogramming that leads to an increase in glucose uptake, higher production of lactate, and increased translocation of GLUT-1 to the cell membrane [34].

Tumor cells usually overexpress GLUT-1 glucose transporter, and this is not expected to be different for fibrosarcoma cells. It is likely that this carrier is the one affected, which can be justified, because, during the evaluation of glucose uptake, cells that were subjected to a negative control (ascorbic acid) exhibited a significant reduction in glucose uptake. It is already known that glucose competes with ascorbic acid for GLUT-1. However, more studies are needed that characterize GLUT transporter activity in this type of cell [35].

Otherwise, the canonical concept of the role of Akt as a protein that promotes proliferation, survival, and cell growth is based on downstream Akt inducing the activation of proteins, such as mTOR, which promotes cell growth in addition to the inactivation of several proapoptotic factors such as BAD, procaspase-9, and transcription factor FKHR (forkhead). Additionally, the activation of Akt promotes the increase of transcription factors that increase the expression of anti-apoptotic genes, such as CREB, as well as direct phosphorylation of NF-kB and HIF-1α.

Another mechanism through which Akt promotes survival is through the inactivation of the p53 tumor suppressor gene [36]. Therefore, Akt activation has been implicated in several types of neoplasms, such as breast [37], colorectal, and prostate [38] cancers, among others. However, this canonical concept is being re-evaluated, and a new emerging concept has arisen, whereby Akt is proposed to have a dual role between tumor growth and the promotion of premature senescence [39].

It has recently been described that the activation of Akt might be related to an increase in glucose in the medium, a process that induces ROS production and is characteristic of hyperglycemia mechanisms, where, if glucose is unable to enter the cell, either by decreasing the translocation of GLUT-1 or by any other mechanism, its extracellular accumulation will allow for the activation of Akt and the induction of premature senescence [40].

Premature cellular senescence is an irreversible state of cell growth arrest that is characterized by a set of physicochemical changes and cellular functions [41]. Currently, it is believed to be mediated by the production of ROS and the deprivation of the nutrients necessary for the survival of the tumor cell [42] under conditions of Akt activation. For example, when Akt and its main downstream modulator, mTOR, are activated under through an increase in ROS or decrease in glucose, cells then become more sensitive to cell death that is induced by deprivation or decreased glucose uptake and ROS overproduction [43]. 

While considering the results that were obtained and the information outlined in previous paragraphs, it is very likely that 6-shogaol induces premature senescence through increased ROS production, and that this is related to the activation of Akt; similarly, the recently described mechanisms suggest that the resulting decrease in glucose uptake makes the cells more sensitive to the induction of ROS-mediated apoptosis, even if the order in which they are triggered is not yet clear. Undoubtedly, research in this field includes a large number of possibilities that should be explored.

## 4. Materials and Methods 

### 4.1. Cell Cultures and Reagents

The fibrosarcoma cell line (HT1080) was obtained from American Type Culture Collection (ATCC-CCL-12), and the human periodontal-derived ligament fibroblast (HPdLF) line from Lonza Ref. CC-7049 (Walkersville, MD, USA). Both cells lines were maintained in complete DMEM Dulbecco’s modified Eagle medium that was supplemented with 10% fetal bovine serum (Lonza, Walkersville, MD, USA) and a 1% antibiotic–antimycotic cocktail, and at 37 °C and 5% CO_2_. 6-Shogaol was obtained from Sigma-Aldrich (St. Louis, MO, USA) [13].

### 4.2. Viability Assay 

Cell viability was assessed while using a MTT assay [44]. Briefly, 1000 cells/well were seeded in a 96-well plate in triplicates. The cells were treated for 24 h according to previous reports [24], with 2.5–150 µM 6-shogaol at 37 °C and 5% CO_2_. Upon conclusion of the treatment, the medium containing 6-shogaol was replaced with 100 µL of fresh DMEM and then incubated with 10 µL of MTT reagent for 2 h at 37 °C, followed by 10 µL detergent reagent at room temperature overnight. Viability was measured at 570 nm while using a FLUOstar microplate reader (Omega, Offenburg, Germany). 

### 4.3. ROS Production Assay

A dihydroethidium (DHE) probe was used to quantify the levels of superoxide anion O_2_^−^ [13]. Cells were seeded at 50,000 cells/well in a 24-well plate in triplicates. Cells were treated for 24 h with 30–70 µM 6-shogaol at 37 °C and 5% CO_2_. The cells were then incubated with 1.5 µL of DHE dye for 30 min. at 37 °C and 5% CO_2_. Mean intensity fluorescence was measured at 535/610 nm using a FLUOstar microplate reader (Omega, Offenburg, Germany). Hydrogen peroxide was used as a positive control. *N*-acetylcysteine was used as negative control in co-treatment with 6-shogaol to measure the recovery from death induced by ROS.

### 4.4. Glucose Uptake Evaluation

A 2-NBDG (2-(*N*-(7-nitrobenz-2-oxa-1,3-diazol-4-yl)amino)-2-deoxyglucose) probe was used to quantify glucose uptake. The cells were treated as previously described. Briefly, the cells were trypsinized, centrifugated, and resuspended in 200 µL of staining buffer (1× PBS and 50 µM 2-NBDG). After 30 min. of incubation, cells were washed twice with 200 µL of 1× PBS. Mean intensity fluorescence of glucose uptake was measured with a green laser (430 nm) while using a Flow Cytometer Guava easyCyte (Merck Millipore, Darmstadt, Germany) [45].

### 4.5. Western Blot Analysis 

A total of 150,000 cells were harvested in lysis buffer (50 M Tris-HCl, 0.1 M NaCl, 1 mM EDTA (Ethylenediamine tetraacetic acid), 10 mM MgCl_2_, 1% Triton X-100, and protease and phosphatase inhibitor cocktail). Equal concentrations of total cell lysate were resolved by 10% SDS-PAGE and then transferred to a polyvinylidene fluoride membrane (PVDF). Nonspecific binding sites were blocked with BSA (10%), followed by an incubation with primary antibodies for anti-mTOR, anti-phospho-mTOR-S2448, anti-PTEN, anti-phospho-PTEN-S380/T382/383, anti-Akt, and anti-phospho-Akt-Ser473. Protein complexes were detected with peroxidase-conjugated secondary antibodies and enhanced with chemiluminescence reagent (Thermo Scientific, Carlsbad, Canada) [46]. The films were exposed to X-ray reagents. 

### 4.6. Akt and Phospho-Akt Expression Analysis by Flow Cytometer

A total of 50,000 cells/well were seeded in a 24-well plate. The cells were treated for 24 h with 10–50 µM 6-shogaol concentrations at 37 °C and 5% CO_2_. After the treatments were complete, cells were fixed with 1.6% formaldehyde for 10 min. Subsequently, cells were permeabilized with methanol for 1 h. The treatments were replaced with two changes of 600 µL staining medium (1× PBS and 2% BSA) and then incubated with a different antibody. The following human antibodies were from Cell Signaling: anti-mTOR (#2972), anti-phospho-mTOR-S2448 (#2971), anti-PTEN (#9552), anti-phospho-PTEN-S380/T382/383 (#9554), anti-Akt (#9272), anti-phospho-Akt-Ser473 (#9271), anti-GAPDH (14C10), and rabbit mAB (#2118) (Danvers, MA, USA). Anti-β-actin was from ABCAM reference (#ab8227) (Cambridge, MA, USA) and was used as the loading control. The secondary antibodies used were either anti-mouse Alexa 488 (1:400) and anti-rabbit Alexa 488 (1:400). Mean intensity fluorescence was measured with a green laser (430 nm) while using Flow Cytometer Guava easyCyte (Merck Millipore, Darmstadt, Germany). Incubations with only secondary antibody were used as the negative controls [47].

### 4.7. Statistical Analysis 

Data are presented as the mean ± SD of at least three independent experiments and they were analyzed by two-way ANOVA followed by a post hoc Dunnett’s test. Data were classified at different levels of significance corresponding to *p* < 0.05 (*), *p* < 0.01 (**), and *p* < 0.001 (***). The results were subject to statistical analysis while using GraphPad Prism 7 (Graph Pad Software Inc, San Diego, CA, USA) [48].

## 5. Conclusions

6-Shogaol showed different effects when used to treat either HT1080 tumor cells or fibroblasts. The decrease in HT1080 viability was associated with the increase in ROS that was caused by treatment with 6-shogaol, unlike fibroblasts, which showed no significant changes. The increase in ROS was attenuated by NAC. It is suggested that in the tumor model (HT1080), 6-shogaol induces premature senescence through increased ROS production, the activation of Akt/mTOR, and the decrease in glucose uptake. Taken together, these effects can make the cells more sensitive to the induction of apoptosis by mechanisms that have not yet been completely described. In future, it will be necessary to evaluate other proteins of the signaling pathway (PI3K/Akt/mTOR) to check the modulation efficacy of pathway activation in inducing apoptosis.
